# Angelica gigas ameliorate depression-like symptoms in rats following chronic corticosterone injection

**DOI:** 10.1186/s12906-015-0746-9

**Published:** 2015-07-03

**Authors:** Bombi Lee, Bongjun Sur, Insop Shim, Hyejung Lee, Dae-Hyun Hahm

**Affiliations:** Acupuncture and Meridian Science Research Center, College of Korean Medicine, Kyung Hee University, 26 Kyungheedae-ro, Dongdaemun-gu, Seoul 130-701 Republic of Korea; BK21 PLUS Korean Medicine Science Center, College of Korean Medicine, Kyung Hee University, Seoul, 130-701 South Korea

**Keywords:** Corticosterone, Depression, Anxiety, Tyrosine hydroxylase, Brain-derived neurotrophic factor, *Angelica gigas*

## Abstract

**Background:**

Repeated injection of corticosterone (CORT) induces dysregulation in the hypothalamic-pituitary-adrenal (HPA) axis, resulting in depression. We examined the effects of *Angelica gigas* extract (AGN) treatment in a rat model of depressive and anxiety-like behaviors, induced by chronic CORT exposure.

**Methods:**

Male rats received 10, 20, or 50 mg/kg AGN (i.p.) 30 min prior to a daily injection of CORT for 21 consecutive days. Activation of the HPA axis in response to the repeated CORT injections was confirmed by measuring serum levels of CORT and the expression of corticotropin-releasing factor in the hypothalamus.

**Results:**

Daily AGN administration significantly reversed the depression and anxiety-like behavioral abnormalities. It also blocked increases in tyrosine hydroxylase expression in the locus coeruleus, and suppressed the decreased expression levels of brain-derived neurotrophic factor (BDNF) and its receptor TrkB mRNAs in the hippocampus.

**Conclusions:**

These findings indicate that administration of AGN prior to high-dose exogenous CORT significantly improved helpless behaviors, possibly by modulating the central noradrenergic system and regulation of BDNF expression in rats. Thus, AGN may be a useful agent for the treatment or alleviation of psychiatric disorders associated with depression and anxiety disorders.

## Background

Depression is a common psychiatric disorder that causes problems for humans worldwide because of its substantial association with disabilities; it can even be life-threatening [1]. In recent years, chronic exposure to stressful life events has become an established and important risk factor for the development and maintenance of many psychological or helpless conditions in humans including depression and anxiety [2]. This has supported the notion that dysregulation of the hypothalamic-pituitary-adrenal (HPA) axis may be involved in the pathogenesis of psychologically related disorders, such as depression [3]. Chronic stress results in dysregulation of the HPA axis in the neuroendocrine system, as evidenced by observations that elevated circulating corticosterone (CORT) levels disrupt the circadian regulation of CORT secretion and the glucocorticoid (GC) receptor-negative feedback circuit [4]. This negative feedback in the hippocampus is impaired in major depression patients, resulting in hyperactivity of the HPA axis and increased levels of humeral corticosteroids [5, 6]. Thus, patients with Cushing’s disease or those undergoing long-term pharmacotherapy with GC exhibit an extremely high rate of depression [7]. Stimulation and sustained actions of the HPA axis is attenuated via the negative feedback action of circulating GC following exogenous CORT administration, and this is closely associated with the development of psychosomatic disorders, which produce serious changes in affective behavior that are indicative of, or consistent with, depressive-like symptoms [8, 9]. Activation of the HPA axis by high-dose CORT administration increases depression-like behavior in rodents, as indicated by a significant decrease in sucrose consumption [8], alterations in a variety of neurotransmitters, and increases in immobility time of forced swimming test (FST) [10, 11]. However, this can be significantly reversed by antidepressants and acupuncture treatment [12–14].

The dried radix of *Angelica gigas*, termed “Korean Dang Gui”, and generally known as “Korean angelica”, is a popular medicinal plant in Korea. The plant has been used traditionally in the treatment of psychosomatic diseases, such as depression via its hemogenic, health-promoting activities, analgesic, and sedative activities in Korean herbal prescriptions [15, 16]. It has multiple pharmacological activities, including anticancer, antibacterial, antiplatelet aggregation, antinematodal activities, and antioxidant properties [15]. Additionally, several studies have indicated that *Angelica gigas* demonstrated anxiolytic and anti-inflammatory effects [17]. Recently, some studies have shown that angelica essential oil exhibited anxiolytic-like effects in three murine tests of anxiety [18], and Japanese angelica root extract reversed stress-induced decrease in pentobarbital sleep through central noradrenergic or GABA_A_ receptors in mice [19]. Although a brief report on the anti-inflammatory activity and anxiolytic-like effects of *Angelica gigas* has been published [17, 18], it is currently unknown whether *Angelica gigas* extract (AGN) can improve the depression- and anxiety-like symptoms induced by repeated CORT injections in rats.

The aim of the present study was to investigate the medicinal impacts of AGN on chronic stress-induced depression- and anxiety-related symptoms in an animal model using behavioral and neurobiological methodologies. Using the forced swimming test (FST) and elevated plus maze (EPM) test, the administration of AGN was evaluated for its efficacy in alleviating depression- and anxiety-like behavior in rats that were repeatedly exposed to exogenous CORT. Additionally, the underlying neurobiological mechanism of these behaviors was investigated through an evaluation of the central noradrenergic system and the regulation of BDNF signaling via TrkB expression in brains of rats following repeated CORT administration.

## Methods

### Animals

Adult male Sprague-Dawley (SD) rats weighing 260-280 g were obtained from Samtako Animal Co. (Seoul, Korea). Animals were maintained on a 12-h light/dark cycle (lights on at 7: 00 a.m., lights off at 7: 00 p.m.) under controlled temperature (22 ± 2°C) and humidity (55 ± 15 %), and they were given standard diet and water during the experiments. The rats were housed in a limited access rodent facility with up to five rats per polycarbonate cage. The animal experiments were conducted in accordance with the National Institutes of Health *Guide for the Care and Use of Laboratory Animals* (NIH Publications No. 80-23), revised in 1996, and were approved by the Kyung Hee University Institutional Animal Care and Use Committee. The animals were allowed 1 week to acclimatize themselves to the housing conditions before the beginning of the experiments. The effects were made to minimize the number and suffering of animals.

### Preparations of methanol extract of Angelica gigas and drugs

Dried roots of *Angelica gigas* was purchased from Jongdo Pharmacy Co., Ltd. (Seoul, Korea). A voucher specimen of *Angelica gigas* has been deposited at the herbarium located at the College of Korean Medicine, Kyung Hee University (No. KH-AG01 for AG). Two hundred grams of *Angelica gigas* were cut into small pieces and extracted three times with 2 L of 80 % methanol by sonication in a reflux condenser for 24 h at room temperature (25 ± 2 °C). The extracted solutions were combined, filtered through Whatman No. 1 filter paper, and concentrated using a rotary vacuum evaporator (EYELA CCA-110, Tokyo Rikakikai Co., Tokyo, Japan) and dried with a freeze dryer (EYELA FD-800). The collection rete of the final methanol extracts was 23.14 (w/w). Corticosterone was purchased from Sigma-Aldrich Chemical Co. (St Louis, MO, USA).

### Experimental groups

This study was designed to explore the efficacy of AGN administration for healing repeated CORT-induced depression- and anxiety-like behaviors in an animal model using behavioral and neurobiological methodologies. Different groups of rats, seven animals per group, were used for drug treatment and tests. Doses were calculated as mg/kg body weight. All the experimental animals including control and drug-treatment groups were subjected to administration. The aim of food withdrawn prior to drug administration was just to ensure the bioavailability of drug. In other time periods, all animals had free access to food. The standard dose of AGN in rat was based on previous study [17]. In addition, considering the long-term treatment used in the present study, the doses of 10, 20, 50 mg/kg every 24 h were selected. The procedure and dose of CORT administration was performed as described in Mao et al. [20]. CORT (40 mg/kg), which was dissolved in absolute ethanol and subsequently diluted in water to the final concentration of 10 % ethanol, was administrated subcutaneously (s.c) in a volume of 1 ml/kg once daily for 21 days [8, 21]. AGN (10, 20, 50 mg/kg) and the positive drug fluoxetine (15 mg/kg, FLX, Fluoxetine hydrochloride; Eli Lilly and Company, Basingstoke, Hampshire) were administered by intraperitoneally (i.p.) in a volume of 10 ml/kg 30 min prior to the CORT injection for 21 days. This CORT dose was selected because it induces serum levels of the steroid comparable to those elicited by substantial stress [22]. As a vehicle control, animal in the SAL group were subcutaneously given the equivalent volumes in saline to the final concentration of 10 % ethanol in a volume of 10 ml/kg. The CORT and saline injections were given in the morning between 9 and 10 am once daily for 21 consecutive days. The SAL group and CORT group also received saline instead of AGN as a vehicle control in an equal volume for a period of 21 days. All drugs were freshly prepared right before every experiment. The following parameters were measured to monitor the effects of the development of psychosomatic disorders by exogenous CORT administration: changes of body weight gains (at the beginning step of exogenous CORT administration), and serum CORT levels (after repeated CORT-induced depression- and anxiety-like symptoms).

### Sucrose preference test

The sucrose preference test was employed herein to evaluate anhedonia, one of the core symptoms of major depression in humans. The test was performed as described by Mao et al. [20]. Before the test, the rats were trained to adapt to sucrose solution (1 % w/v) by placing two bottles of sucrose solution in each cage for 24 h prior to the start of the experiment; then one of the bottles was replaced with water for 24 h. They were exposed to 1 % sucrose solution for 24 h period in their home cages without any food or water available. After the adaptation procedure, the rats were deprived of water and food for 10 h. The sucrose preference test was conducted at 10:00 a.m. The rats were housed in individual cages and given free access to the two bottles containing 100 mL of sucrose solution (1 %, w/v) and 100 mL of water, respectively. Sucrose and water consumption was measured for the period of 3 h by weighing pre-weighed bottles at the end of the test. Sucrose preference was measured by calculating the proportion of sucrose consumption out of total consumption of liquid and was recorded once three days during the experiment for 21 consecutive days.

### Corticosterone (CORT) and brain-derived neurotrophic factor (BDNF) analysis

After CORT injection for 21 days, CORT concentration in blood, and BDNF concentration in brain tissue were determined. Animals were killed by decapitation one day after behavioral measurements. To avoid fluctuations on hormone levels due to circadian rhythm, animals were bled at 12:00-1:00 p.m. on the day of sacrifice. Blood samples were collected for the determination of serum CORT levels. For this, the unanesthetized rats were rapidly decapitated, and blood was quickly collected via the abdominal aorta. The hippocampus or medial prefrontal cortex were rapidly removed from the rat brains in randomized order. Blood was centrifuged at 4000 g for 10 min, and serum was collected and stored at -70 °C until use. The CORT concentration was measured by a competitive enzyme-linked immunoassay (ELISA) using a rabbit polyclonal CORT antibody (Abcam Corticosterone ELISA kit; Cambridge, MA, USA) according to the manufacturer’s protocol. The brain tissue samples were stored at -80 °C until use. Hippocampus or medial prefrontal cortex were homogenized in a lysis buffer containing 137 mM NaCl, 20 mM Tris (pH 8.0), 1 % NP40, 10 % glycerol, 1 mM PMSF, 10 mg/ml aprotinin, 1 mg/ml leupeptin and 0.5 mM sodium vanadate. Homogenization was carried out on ice using a tissue homogenizer and incubated for 1 min at 4 °C with shaking. Homogenates were centrifuged and supernatants were collected. The BDNF concentration was measured by a competitive enzyme-linked immunoassay (ELISA) using a mouse monoclonal BDNF antibody (Novus biologicals BDNF kit; Novus Biologicals, LLC., Littleton, CO, USA) according to the manufacturer’s protocol. Samples (or standard) and conjugate were added to each well, and the plate was incubated for 1 h at room temperature without blocking. After wells were washed several times with buffers and proper color developed, the optical density was measured at 450 nm using an ELISA reader (MutiRead 400; Authos Co., Vienna, Austria).

### Forced swimming test (FST)

The FST used was the same as described in detail elsewhere [23], with some modification. Briefly, the test was done by placing a rat in a transparent Plexiglas cylinder (20 cm diameter × 50 cm height) was filled up to a depth of 30 cm with water at 25°C. At this depth, rats could not touch the bottom of the cylinder with their tails or hind limbs. Two swimming sessions were conducted: an initial 15 min pretest, followed by a 5 min test 24 h later. Animals were subjected to 5 min of forced swim, and escape behaviors (climbing and swimming) were determined. The animals’ behavior was continuously recorded by experimenter-manual scoring during the testing session with an overhead video camera to tape behavior for later manual scoring. Immobility behavior was calculated as the length of time in which the animal did not show escape responses (e.g., total time of the test minus time spent in climbing and swimming behaviors). The rats were judged to be immobile when it remained in the water without struggling and was making only those movements necessary to keep its head above water. Climbing behavior was defined as upward-directed movements of the forepaws alone the side of the swim chamber and swimming behavior was considered as movements throughout the swim chamber including crossing into another quadrant.

### Elevated plus maze test (EPM)

The EPM test is a widely used behavioral test to assess anxiogenic or anxiolytic effects of pharmacological agents. Animals conduct anxiety-like behaviors usually show the reductions both in the number of entries and in the time spent in the open arms, along with an increase in the amount of time spent in the closed arms in the EPM. The elevated plus test was conducted. This apparatus consisted of two open arms (50 × 10 cm each), two closed arms (50 × 10 × 20 cm each) and a central platform (10 × 10 cm), arranged in a way such that the two arms of each type were opposite to each other. The maze was made from black Plexiglas and elevated 50 cm above the floor. Exploration of the open arms was encouraged by testing under indirect dim light (2 × 60 W). During a 5-min test period, the following parameters were recorded: 1) number of open arm entries, b) number of closed arm entries, c) time spend in open arms, and d) time spent in closed arms. Entry by an animal into an arm was defined as the condition in which the animal has placed its four paws in that arm. The behavior in the maze was recorded using a video camera mounted on the ceiling above the center of the maze and relayed to the S-MART program (PanLab, Barcelona, Spain). Anxiety reduction, indicated by open arm exploration in the EPM, was defined as an increase in the numbers of entries into the open arms relative to total entries into either open or closed arm, and an increase in the proportion of time spent in the open arms relative to total spending time in either open or closed arm. Total arm entries were also used as indicators of changes in locomotor activities of the rats.

### Open field test (OFT)

Prior to forced swimming test, the rats were individually housed in a rectangular container that was made of dark polyethylene (60 × 60 × 30 cm) to provide best contrast to the white rats in a dimly lit room equipped with a video camera above the center of the room, and their locomotion (animal’s movements) and exploratory behavior were then measured. The locomotion indicated by the time and the distance of movements was monitored by a computerized video-tracking system using S-MART program (PanLab Co., Barcelona, Spain). The open field maze was divided into two zones, central and peripheral zone, using the square drawn on the maze. The area was divided into 16 squares of 15 × 15 cm by painted white lines. The rats was placed in one corner of the open filed and its activity during the subsequent 5 min was assessed. Locomotion (central zone crossing) and time spent in central and peripheral zone were observed [24].

### Immunohistochemistry of corticotropin-releasing factor (CRF) and tyrosine hydroxylase (TH)

For immunohistochemical studies, the seven rats in each groups were deeply anesthetized with sodium pentobarbital (80 mg/kg, by intraperitoneal injection) and perfused through the ascending aorta with normal saline (0.9 %) followed by 300 ml (per rat) of 4 % paraformaldehyde in 0.1 M phosphate-buffered saline (PBS). The brains were removed in a randomized order, post-fixed over-night, and cryoprotected with 20 % sucrose in 0.1 M PBS at 4 °C. Coronal sections 30 μm thick were cut through hypothalamus and locus coeruleus (LC) using a cryostat (Leica CM1850; Leica Microsystems Ltd., Nussloch, Germany). The sections were immunostained for CRF and TH expression using the avidin-biotin-peroxidase complex (ABC) method. Briefly, the sections were incubated with primary goat anti-CRF antibody (1:500 dilution; Santa Cruz Biotechnology Inc., California, CA, USA) and sheep anti-TH antibody (1:2000 dilution; Chemicon International Inc., Temecular, CA, USA) in PBST (PBS plus 0.3 % Triton X-100) for 72 h at 4°C. The sections were incubated for 120 min at room temperature with secondary antibody. The secondary antibodies were obtained from Vector Laboratories Co. (Burlingame, CA, USA) and diluted 1:200 in PBST containing 2 % normal serum. To visualize immunoreactivity, the sections were incubated for 90 min in ABC reagent (Vectastain Elite ABC kit; Vector Labs. Co., Burlingame, CA, USA), and incubated in a solution containing 3,3′-diaminobenzidine (DAB; Sigma-Aldrich Chemical Co., St. Louis, MO, USA). Finally, the tissues were washed in PBS, followed by a brief rinse in distilled water, and mounted individually onto slides. Images were captured using the AxioVision 3.0 imaging system (Carl Zeiss, Inc., Oberkochen, Germany) and processed using Adobe Photoshop (Adobe Systems, Inc., San Jose, CA, USA). The sections were viewed at 200 × magnification, and the numbers of CRF and TH labeled cells was quantified in the hypothalamus and LC. CRF- and TH-labeled cells were counted by an observer blinded to the experimental groups. The counted sections were randomly chosen from equal levels of serial section along the rostral-caudal axis. The cells within the hypothalamus and LC areas were counted on each of the five sections per animal. Counting the immunopositive cells were performed within the square (150 × 150 μm^2^), anatomically localized in hypothalamus (Bregma -1.80 mm) and LC (Bregma -9.96 mm) sections per rat brain according to the stereotactic rat brain atlas of Paxinos and Watson [25]. Immunohistochemistry analysis of tissue samples from 3 different animals was enough to show the changes of protein markers at molecular levels, as published in our previous studies [22]. Distinct brown spots indicating CRF- and TH-immunopositive cells were observed in the hypothalamus and LC. The CRF and TH-immunopositive cells were only counted when their densities reached a defined darkness above the background level. The brightness and contrast of all images were not adjusted to remove any subjective selection of immunoreactive cells.

### Total RNA preparation and RT−PCR analysis

The expression levels of BDNF and TrkB mRNAs were determined by the reverse transcription**-**polymerase chain reaction (RT**-**PCR). The brain hippocampus was isolated from three rats per group. After decapitation, the brain was quickly removed and stored at -80 °C until use. The total RNA was prepared from the brain tissue using a TRIzol® reagent (Invitrogen Co., Carlsbad, CA, USA) according to the supplier’s instruction. Complementary DNA was first synthesized from total RNA using reverse transcriptase (Takara Co., Shiga, Japan). PCR was performed using a PTC-100 programmable thermal controller (MJ Research, Inc., Watertown, MA, USA). The operating conditions were as follows: for glyceraldehydes-3-phosphate dehydrogenase (GAPDH), 30 cycles of denaturation at 95 °C for 30 s, annealing at 58 °C for 30 s, and extension at 72 °C for 30 s; for BDNF, 27 cycles of denaturation at 95 °C for 30 s, annealing at 57 °C for 30 s, and extension at 72 °C for 30 s; for TrkB, 38 cycles of denaturation at 95 °C for 30 s, annealing at 59 °C for 30 s, and extension at 72 °C for 30 s. All primers were designed using published mRNA sequences of those cytokines and a primer designing software, Primer 3, offered by the Whitehead Institute for Biomedical Research (Cambridge, MA, USA; http://primer3.ut.ee) on the website. The following sequences were used: for GAPDH (409 bp), (forward) 5′-ATC CCA TCA CCA TCT TCC AG-3′ and (reverse) 5′-CCT GCT TCA CCA CCT TCT TG-3′; for BDNF (153 bp), (forward) 5′-CAG GGG CAT AGA CAA AAG-3′ and (reverse) 5′-CTT CCC CTT TTA ATG GTC-3′; for TrkB (347 bp), (forward) 5’-TGG GAC GTT GGG AAT TTG GTT-3’ and (reverse) 5’-CAG CCG TGG TAC TCC GTG TG-3’. The PCR products were separated on 1.2 % agarose gels and stained with ethidium bromide. The density of each band was quantified using an image-analyzing system (i-Max™, CoreBio System Co., Seoul, Korea). The expression levels were compared each other by calculating the relative density of target band, such as BDNF and TrkB, to that of GAPDH.

### Statistical analysis

All measurements were performed by an independent investigator blinded to the experimental conditions. Results in figures are expressed as mean ± standard error of means (SE). Differences within or between normally distributed data were analyzed by analysis of variance (ANOVA) using SPSS (Version 13.0; SPSS, Inc., Chicago, IL, USA) followed by Tukey’s *post-hoc* test. Statistical significance was set at *p* < 0.05.

## Results

### Effects of AGN on CORT-induced body weight loss, increase of serum CORT levels and reduction in consumed sucrose intake

**Fig. 1:**
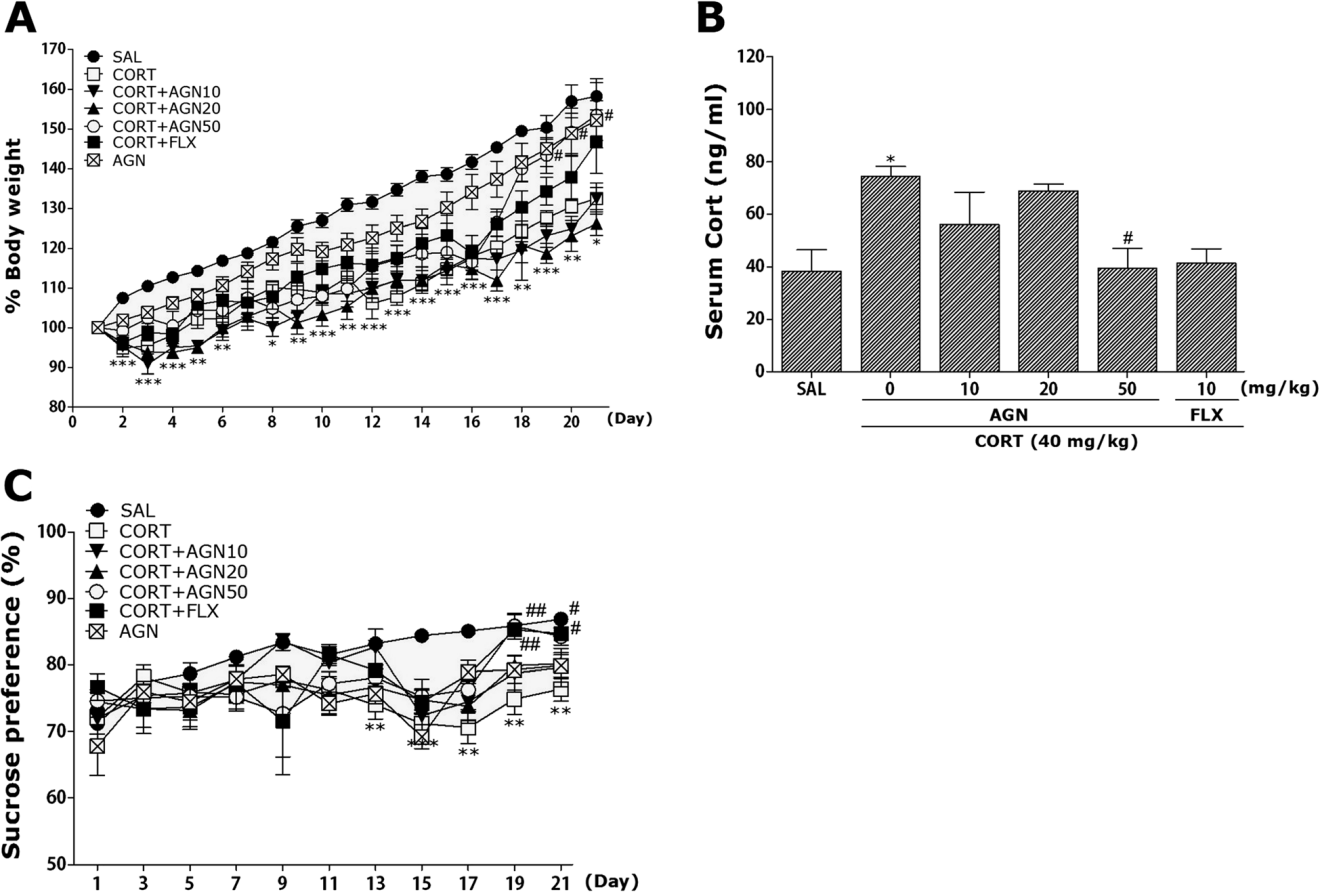
Effect of AGN administration on body weights gain (**a**) serum corticosterone levels (**b**), and sucrose intake (**c**) of the rats under chronic CORT injection for 21 consecutive days. ^*^ *p* < 0.05, ^**^ *p* < 0.01, ^***^ *p* < 0.001 *vs*. SAL group; ^#^ *p* < 0.05, ^##^ *p* < 0.01 *vs*. CORT group

Rats exposed to the repeated administration of exogenous CORT begin to lose body weight on the first day of CORT injections and this body weight loss is sustained for a prolonged period of time without restoration and is even exacerbated in some cases [22]. In the present study, body weight was evaluated daily for 21 days to identify whether the repeated administration of CORT (CORT group) would result in body weight loss (difference between daily weights and starting weight; Fig. 1a). Analysis of the body weight values revealed a significant gradual reduction of body weight gain over 21 days in the CORT group relative to control rats (SAL group). During this period, rats treated with 50 mg/kg AGN exhibited a significant inhibition of the reduction in body weight gain compared to the CORT group (*p* < 0.05 on days 19, 20 and 21). It was that only AGN (50 mg/kg)-treated group did not show any change in body weight when compared with the saline-treated group (*p* = 0.924).

Additionally, the serum CORT levels were measured in each group following the repeated administration of CORT for 21 days. ELISA analysis revealed that CORT administration over 21 days significantly increased serum CORT concentrations by 193.74 % compared to saline-treated rats (Fig. 1b; *p* < 0.05). This indicates that repeated CORT injections were sufficiently stressful despite the evoked CORT response (physiological response) to repeated CORT injections being significantly greater than the response to a single CORT injection (data not shown). In these results, the exogenous CORT-induced depression-like symptoms were exploited to develop a chronic stress model in rats. Daily administration of AGN inhibited the exogenous CORT-induced increase of serum CORT levels compared to the CORT group (*p* < 0.05).

In the present study, sucrose preference in rats was examined once every three days over 21 days to identify whether the repeated administration of CORT resulted in differences in the preference of sucrose compared to saline-treated rats (Fig. 1c). The analysis of sucrose intake revealed a significant gradual reduction in consumed sucrose intake over 21 days in the CORT group compared to the SAL group (*p* < 0.01 on days 13, 17, 19, and 21; *p* < 0.001 on day 15). During this period, rats treated with 50 mg/kg of AGN exhibited a significant inhibition of the reduction in consumed sucrose intake compared to the CORT group (*p* < 0.01 on day 19; *p* < 0.05 on day 21). Fluoxetine, the positive drug, also increased the sucrose preference in the rat exposed to CORT (*p* < 0.01 on day 19; *p* < 0.05 on day 21).

### Effects of AGN on CORT-induced depression-like behaviors

**Fig. 2:**
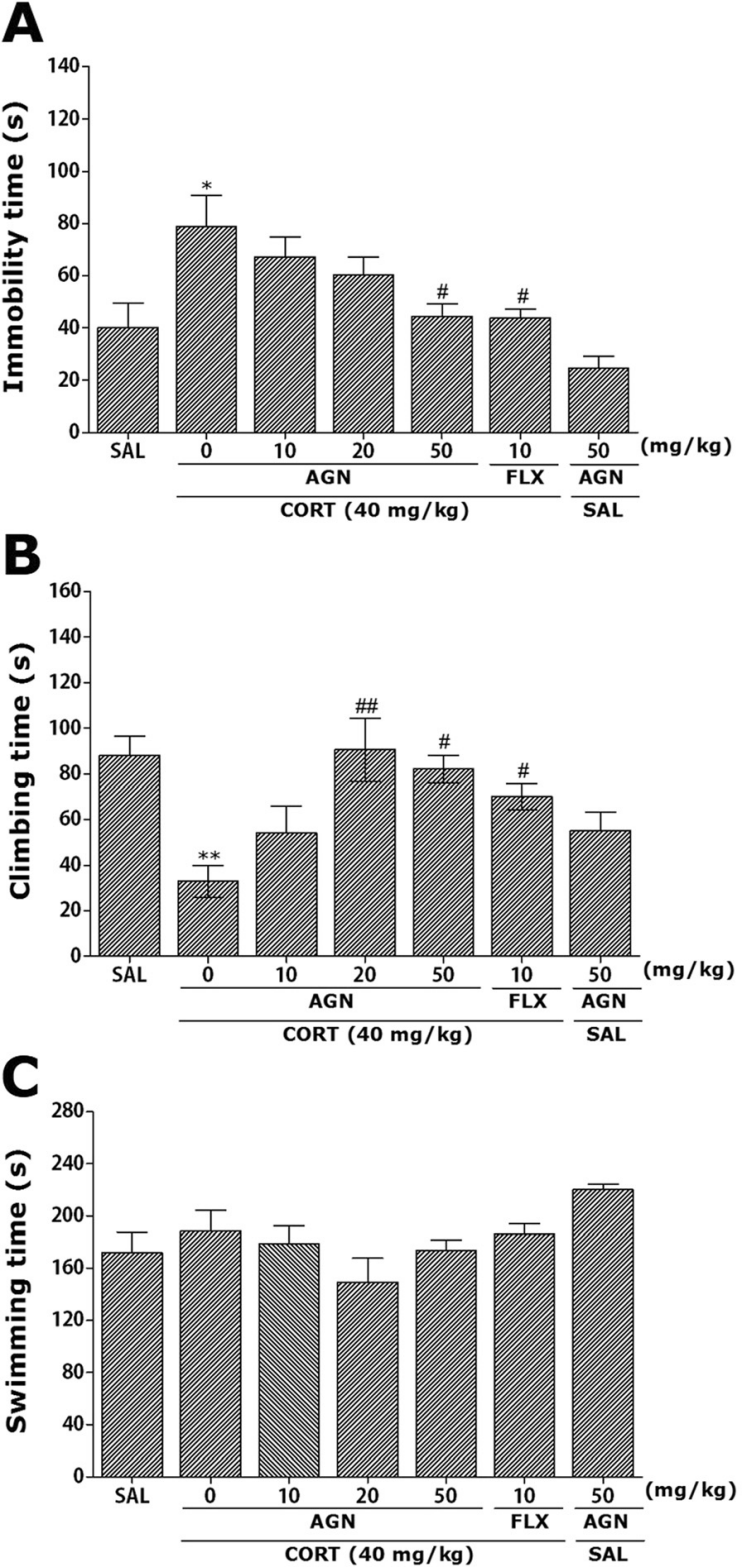
Effect of AGN administration on immobility time (**a**), climbing behavior (**b**) and swimming behavior (**c**) in forced swimming test during chronic CORT injection. ^*^ *p* < 0.05, ^**^ *p* < 0.01 *vs*. SAL group; ^#^ *p* < 0.05, ^##^ *p* < 0.01 *vs*. CORT group

Rats subjected to the repeated administration of exogenous CORT for 21 days exhibited a significant depression-like phenotype, characterized by increased an increased duration of immobility during the FST compared to saline-treated controls (Fig. 2). Rats in the CORT group exhibited more immobility during the FST compared to the SAL group (*p* < 0.05; Fig. 2a). Long-term treatment with AGN (50 mg/kg) for 21 days displayed a significant decrease in durations of immobility during 5 min in the FST compared to CORT group (*p* < 0.05), indicating that the administration of 50 of mg/kg AGN decreases depression-like behaviors. Another key behavior, climbing behavior was also analyzed. Rats in the CORT group exhibited a significant decrease in climbing behavior during the FST relative to the SAL group (*p* < 0.01; Fig. 2b). Furthermore, compared with the CORT group, rats in the CORT + AGN20 (*p* < 0.01) and CORT + AGN50 (*p* < 0.05) groups exhibited a significant restoration of climbing behavior time during 5 min in the FST. It was that only AGN (50 mg/kg)-treated group did not show any change in immobility time and climbing behavior in the FST when compared with the saline-treated group (*p* = 0.513) and (*p* = 0.317). This indicates that CORT administration significantly restores depression-like despair behaviors. However, repeated administration of exogenous CORT over 21 days did not induce significant differences in swimming behaviors among the groups during the FST (*p* = 0.988; Fig. 2c). These results reveal that the recovery of climbing behavior and the reduction in immobility during depression-like behaviors in the CORT + AGN50 group were almost comparable to those of the CORT + FLX group.

### Effects of AGN on CORT-induced anxiety-like behaviors

**Fig. 3:**
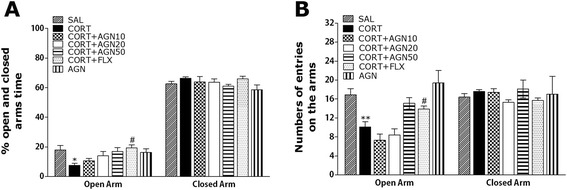
Effect of AGN administration on the percentage of time spent on open and closed arms (**a**) and the numbers of entries into open and closed arms (**b**) in the elevated plus maze during chronic CORT injection. ^*^ *p* < 0.05, ^**^ *p* < 0.01 *vs*. SAL group; ^#^ *p* < 0.05 *vs*. CORT group

**Fig. 4:**
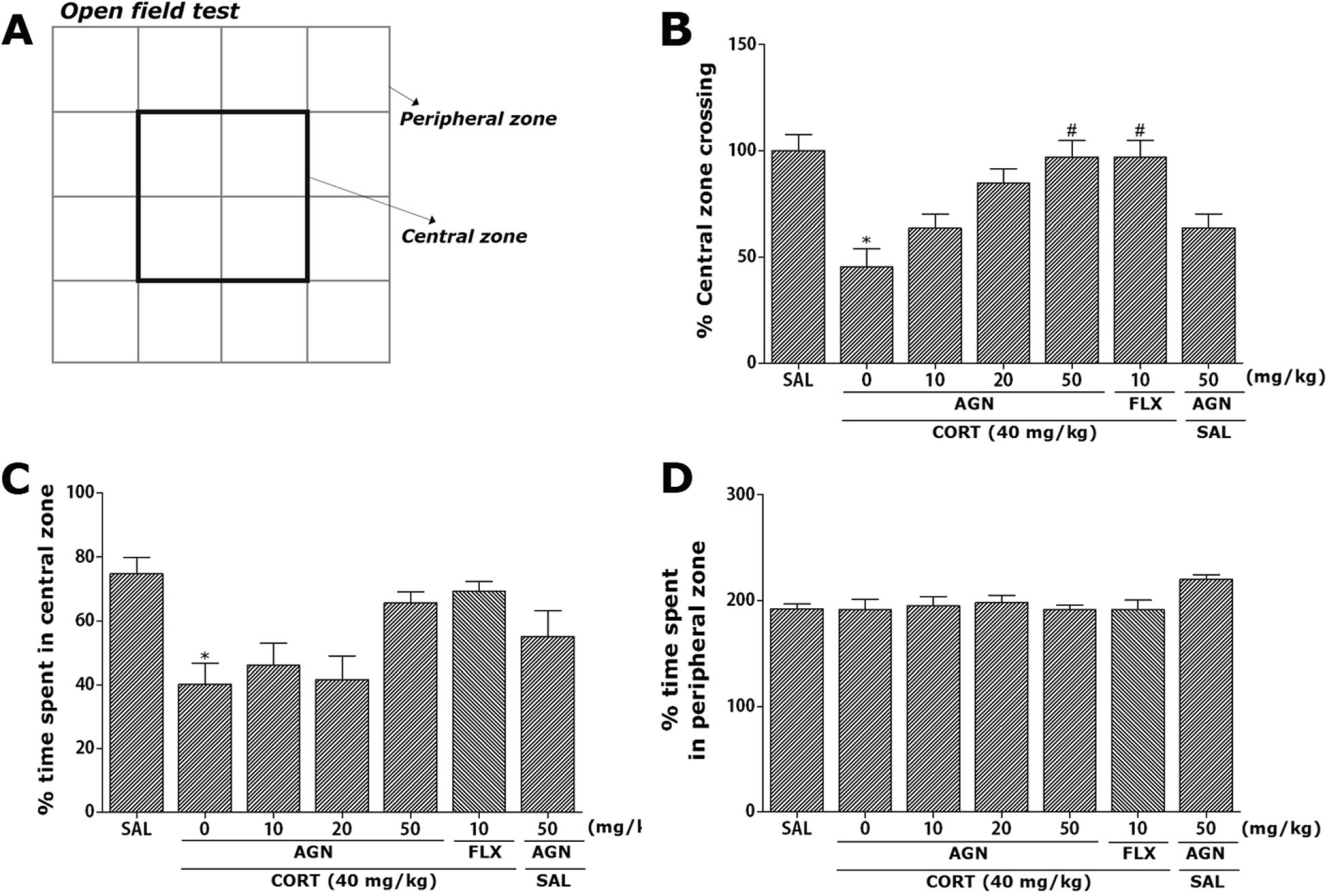
Effect of AGN administration on locomotion and exploratory behavior in the open-field test during chronic CORT injection. Representative photographs are showed the central zone and peripheral zone (**a**). Change in the central zone crossing (**b**) and the time spent in central zone (**c**) and peripheral zone (**d**). ^*^ *p* < 0.05 *vs*. SAL group; ^#^ *p* < 0.05 *vs*. CORT group

The effects of AGN administration on anxiety-like behaviors, characterized by decreases in open-arm exploration in the EPM test, were also investigated (Fig. 3). Post hoc comparisons revealed a significant decrease in the percentage of time spent by rats in the open arms of the maze following the repeated administration of exogenous CORT for 21 days compared to the saline-treated rats (*p* < 0.05). However, compared to the CORT group, rats in the CORT + AGN50 group exhibited a slightly increased restoration of the percentage of time spent in open arms of the maze, which was formerly decreased by CORT-induced anxiety-like behaviors, although the findings were only minimally significant (*p* = 0.071; Fig. 3a). Similarly, post hoc comparisons revealed a significant decrease in the number of entries into the open arms of the maze after the repeated administration of exogenous CORT for 21 days compared to the SAL group (*p* < 0.01). Rats in the CORT + AGN50 group also exhibited a significant restoration in the number of entries into the open arms of the maze compared to the CORT group (*p* < 0.05; Fig. 3b). It was that only AGN (50 mg/kg)-treated group did not show any change in open-arm exploration in the EPM test when compared with the saline-treated group (*p* = 0.495) and (*p* = 0.419). Because no significant differences appeared in the number of closed-arm entries between groups in the EPM test, the observed anxiety-like behaviors of the rats receiving repeated CORT injections are likely not attributable to differences in their locomotor activities (*p* = 0.998; Fig. 4b). AGN administration without the prior repeated administration of exogenous CORT did not elicit anxiolytic or anxiogenic behavior in this study. These results reveal that the increase in the number of entries into the open arms of the maze by the CORT + AGN50 group was almost comparable to those of the CORT + FLX group.

### Effects of AGN on CORT-induced locomotion and exploratory behaviors

Open-field activity was used to evaluate locomotion and exploratory behavior among the rats receiving CORT injections for 21 days (Fig. 4). Rats receiving CORT injections significantly decreased the time spent in central zone with corresponding increase in the time spent in the peripheral zone compared to the SAL group (*p* < 0.05). There was also significant decrease in central zone crossing following chronic CORT exposure (*p* < 0.05). This finding suggests that CORT-treated rats subsequently produce exploration activities that are closely associated with anxiety-like behaviors in the open-field test. However, AGN-treated rats (50 mg/kg) displayed a significant increase in central zone crossings compared to the CORT group (*p* < 0.05), indicating that anxiety-like behaviors in the CORT + AGN50 group was almost comparable to those of the CORT + FLX group.

### Effects of AGN on CORT-induced CRF- and TH-like immunoreactivities

**Fig. 5:**
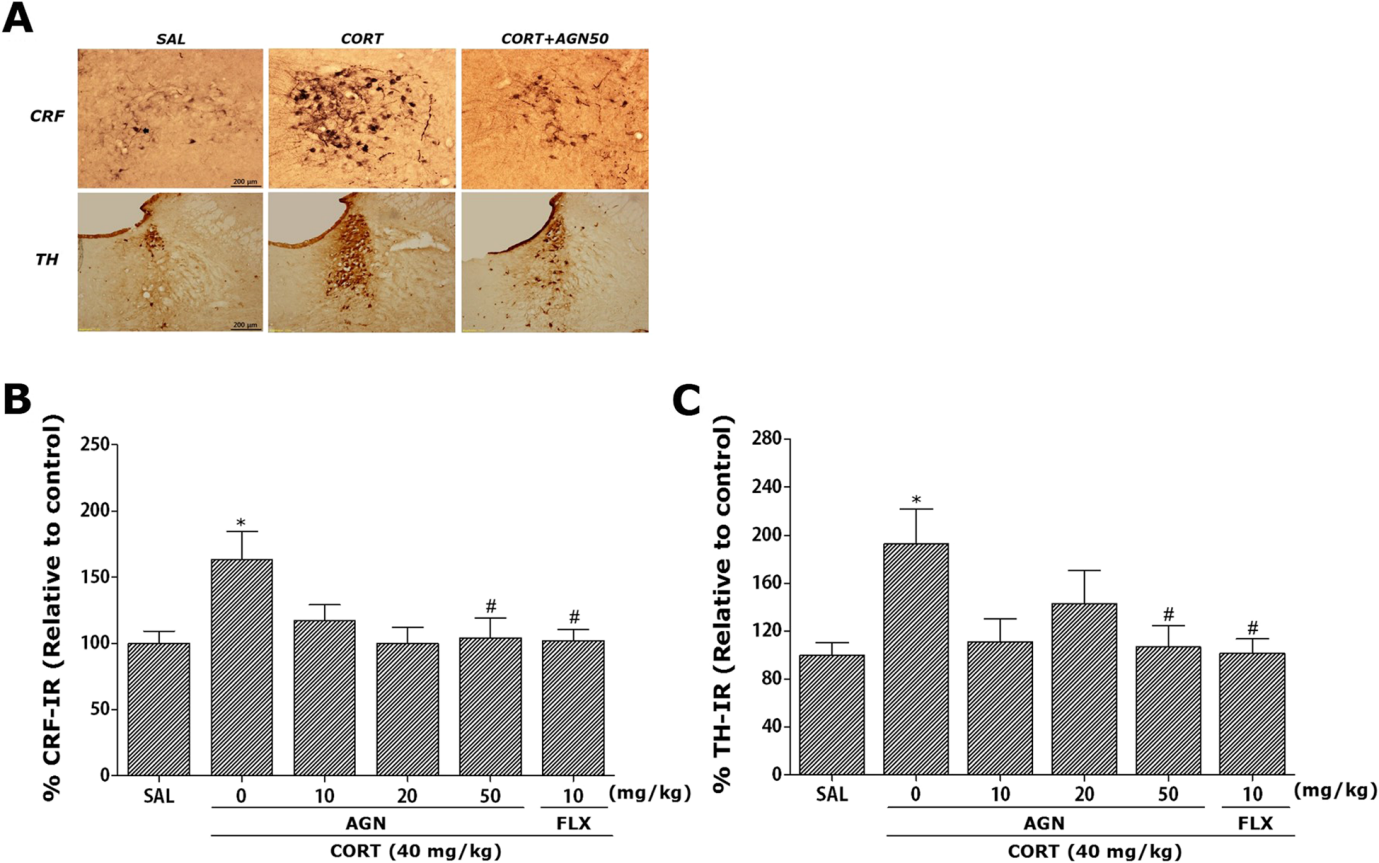
Effects of AGN administration on the mean number of corticotropin-releasing factor (CRF) expression in the paraventricular nucleus (PVN) of the hypothalamus and tyrosine hydroxylase (TH) expression in the locus coeruleus (LC). Representative photographs and the relative percentage values are indicated in (**a**) and (**b** and **c**), respectively. ^*^ *p* < 0.01 *vs*. SAL group; ^#^ *p* < 0.05 *vs*. CORT group

Following the behavioral tasks, CRF-like immunoreactivity was analyzed in the cell bodies of various hypothalamic regions including the paraventricular nucleus (PVN; Fig. 5a). The numbers of CRF-immunoreactive fibers in the PVN of the CORT group were increased by 164.35 %. Analysis of the numbers of CRF-immunoreactive neurons values revealed that rats receiving repeated administration of exogenous CORT exhibited a significant increase of CRF expression compared to the SAL group (*p* < 0.05; Fig. 5b). The number of CRF-immunoreactive neurons was significantly decreased in the PVN region of the CORT + AGN50 group compared to the CORT group (*p* < 0.05). This finding suggests that the increased CRF-immunoreactivity induced by the repeated administration of exogenous CORT was significantly restored by AGN administration and that the number of CRF-immunopositive neurons in the CORT + AGN50 group was closely associated with that in the CORT + FLX group (*p* < 0.05). TH-like immunoreactivity was also analyzed in adrenergic regions including the LC (Fig. 5a). The numbers of TH-immunoreactive fibers in the LC of the CORT group increased to 192.73 %. Analysis of the numbers of TH-immunoreactive neurons values revealed that rats repeatedly exposed to exogenous CORT exhibit a significant increase of TH expression compared to the SAL group (*p* < 0.05; Fig. 5c). The number of TH-immunoreactive neurons significantly decreased in central adrenergic regions of the CORT + AGN50 group relative to the CORT group (*p* < 0.05). This finding suggests that the increased TH-immunoreactivity induced by the repeated administration of exogenous CORT was significantly restored by AGN administration and that the number of TH-immunopositive neurons in the CORT + AGN50 group was closely associated with that in the CORT + FLX group (*p* < 0.05).

### Effect of AGN on CORT-induced decrease of BDNF levels in the hippocampus

**Fig. 6:**
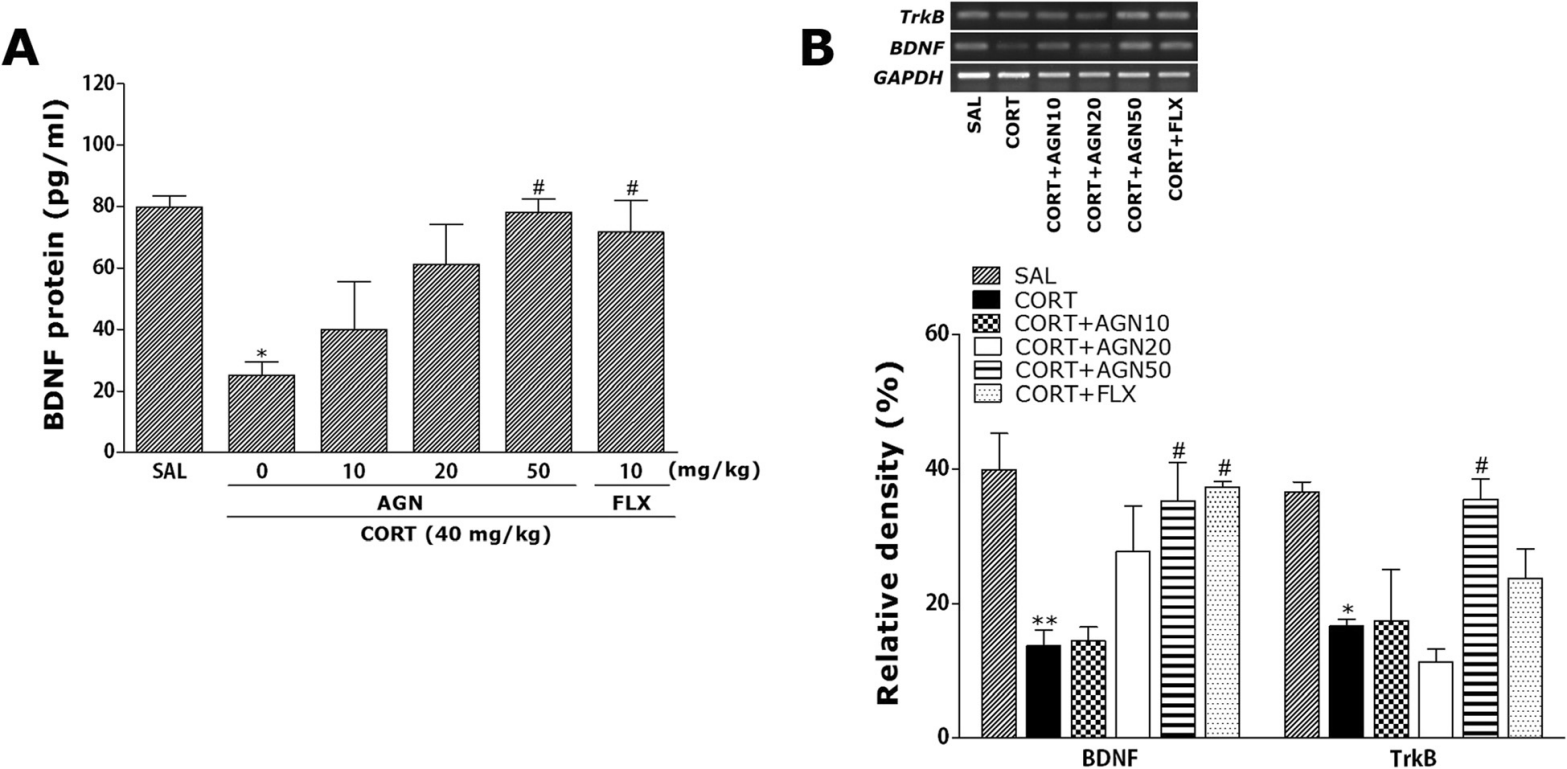
Effect of AGN administration on the expression of (BDNF) and TrkB mRNAs (**a**) and level of BDNF protein concentration (**b**) in rats subjected to chronic CORT injection for 21 consecutive days. The expression levels of BDNF and TrkB mRNAs were normalized to that of glyceraldehyde 3-phosphate dehydrogenase (GAPDH) mRNA as an internal control. ^**^ *p* < 0.01 *vs.* the SAL group; ^#^ *p* < 0.05 *vs.* CORT group

The ELISA analysis demonstrated that repeated CORT injection for 21 days significantly decreased the BDNF concentration in the hippocampus by 31.28 % compared with rats in the non-treated SAL group. The concentration of BDNF in the hippocampus was markedly decreased in the CORT group, as compared to the SAL group (*p* < 0.05; Fig. 6a). Daily administration of AGN showed significantly increased the chronic CORT-induced decrease of BDNF concentration in the hippocampus, as compared to CORT group (*p* < 0.05). It also indicated that the concentration of BDNF in the hippocampus in rats receiving 50 mg/kg AGN administration was almost compatible with the rats receiving 10 mg/kg FLX administration.

### Effect of AGN on CORT-induced expression of BDNF and its receptor, TrkB mRNAs in the hippocampus

To investigate the effects of AGN on the mRNAs expression of BDNF and TrkB, which are two molecules crucial for antidepressant-like behavior and neurogenesis, we conducted RT-PCR analysis after the CORT administration for 21 days (Fig. 6b). Hippocampal expression of BDNF mRNA in the CORT group was significantly decreased compared with that in the SAL group (*p* < 0.01). The decreased expression of BDNF mRNA in the CORT group was significantly restored in the CORT + AGN50 group (*p* < 0.05). Hippocampal expression of TrkB mRNA in the CORT group was also significantly decreased compared with that in the SAL group (*p* < 0.05). The decreased expression of TrkB mRNA in the CORT group was significantly increased in the CORT + AGN50 group (*p* < 0.05). This also indicated that the expression of BDNF mRNA in the hippocampus in rats receiving 50 mg/kg AGN administration was closely associated with that in rats receiving 10 mg/kg FLX administration.

## Discussion

Previous studies on the emotional effects of chronic CORT injections in rodents have produced controversial results [26]. Several studies demonstrated that administration of the exogenous stress hormone CORT increased the probability of depression-like behavior in the FST and tail suspension test (TST), consistent with present results [8, 9]. GCs also reach the brain, where they exert an inhibitory influence, halting HPA axis activity through negative feedback inhibitory regulation [27]. However, if prolonged stress exists, chronically elevated GC levels will occur, impairing the negative feedback inhibition, and finally leading to dysregulated emotional and physiological homeostasis, which are seen in the etiology of depression [28]. Thus, the present study suggests that administration of exogenous CORT may be a useful method for studying the relationship between stress, behaviors, CORT, neurotrophic factors, and depression [29].

Our study is basically intended to test effect of administration of AGN on prevention of chronic CORT-induced depression-like behavior rather the cure. Therefore, in order to test administration of AGN as the therapeutic agent for improve depression and anxiety-like symptom in rats exposed to chronic CORT-induced pathogenesis of psychologically related disorders, the administration of AGN was immediately applied before the CORT injection, and thereby we did examine to evaluate the ability of administration of AGN on prevention as measured by their performance in the FST and the EPM test. Therefore, our results showed that administration of AGN prior to CORT injection might be attributable to changes in behavioral task-related pathways that are modulated by the HPA axis.

Also, an increase in immobility time and reduction of sucrose preference, which are typically observed in depression in animal models, might be due to dysregulation of the HPA axis following the artificial injection of CORT [30]. Sucrose preference reduced, which reflects the symptom of anhedonia (inability to experience pleasure) existing in depressed patients, can be improved by antidepressants [8]. Anhedonia, a core symptom of major depression in humans, is modeled by inducing a decrease in responsiveness to rewards, as reflected by reduced consumption or preference for sweetened solutions [31]. This theory has been supported by several studies in which elevated levels of CORT result in an alteration of HPA axis activity that affected behavioral activity and preference for sucrose [32]. Thus, the present findings revealed that administration of AGN at 50 mg/kg significantly increased preference for sucrose and reversed the anhedonic-like behavior, suggesting antidepressant-like actions of AGN.

Furthermore, the current results are consistent with previous findings showing that repeated CORT injections increase immobility during the FST [21]. Here, the administration of AGN significantly decreased immobility and also increased climbing behaviors during the FST, but there was no effect on swimming, confirming an antidepressant-like activity that did not result in motor function deficits [9, 21]. Our observations of an anxiety-like behavior in rats following CORT exposure are also consistent with previous findings [33]. Several studies have suggested that CORTs are involved in the anxiety-like features that are frequently seen in psychiatric disorders such as depression [34, 35]. In the present study, the administration of AGN prior to chronic CORT injections also significantly reduced anxiety-like behaviors in the EPM test, as indicated by the increase in the number of entries into open arms. Using a chronic CORT-exposed rat model, we provided evidence for the anxiolytic-like potential of AGN, in the EPM test [36]. An open-field test was also performed to rule out any confounding motor impairment that can influence outcomes in many behavioral tests of depression or anxiety [37]. No significant individual difference in locomotor activity was observed between groups, suggesting that the administration of AGN had no effect on sensorimotor performance (data not shown). However, the administration of AGN prior to chronic CORT injections significantly reduced anxiety-like behaviors, as indicated by an increase in the central zone crossing in the open-field test. Accordingly, these results suggest that the observed increase in the central zone crossing in the open-field test was similar to the observed changes in behavioral performance in the EPM test, which is likely to be due to anxiolytic activity [25]. Together, the results obtained from the behavioral studies confirmed that AGN treatment exerted an antidepressant-like effect or anxiolytic action in the rats treated with CORT.

When measured immediately after behavioral testing, a gradual decrease in body weight gain and an increase in serum CORT levels was observed, indicating that the chronic CORT injections were sufficiently stressful [22, 38]. It was observed in our study that chronic administration of high-dose CORT caused an elevation of serum CORT levels in the rats, consistent with the previous studies [39]. Accordingly, in animal models, forced sustaining of high CORT levels can affect animal depression-like symptoms under experimental conditions, and this might be associated with the progression or exacerbation of chronically stressful conditions in humans [39]. Thus, the present study revealed that the behavioral consequences of repeated CORT administration were accompanied by dysregulation of the HPA axis. The administration of AGN significantly restored body weight and decreased the serum CORT levels in the late period of AGN administration, suggesting that this therapy inhibited the HPA axis-associated psychological dysfunction induced by repeated CORT injections. Our results may help to explain why administration of AGN affects the hypothalamus in receiving biochemical and behavioral signals induced by reduced CORT levels in serum. The current data suggest that the CRF circuits in the PVN of the hypothalamus are activated by chronic CORT injections, leading to the observed depressive- and anxiety-like activity in the behavioral tests [9]. These results show that the administration of AGN significantly blocks the increase in CRF immunoreactivity in the PVN. This suggests that anti-depressive and anxiolytic effects following the administration of AGN are closely associated with CRF modulation in the PVN in the hypothalamus and activation of the HPA axis [28].

In the present study, TH immunoreactivity in the LC in response to repeated CORT injections was greater in the CORT group versus the SAL group. These results are consistent with previous reports that depressive- and anxiety-like behaviors induced by chronic stress are the result of alterations in the central noradrenergic system [40]. Moreover, these findings demonstrate that the administration of AGN significantly reduced TH-like immunoreactivity in the LC that was previously activated by chronic CORT injections [41]. Thus, these results suggest that administration of AGN may indirectly alter catecholamine synthesis in the brain, producing pharmacological effects [42]. This would suggest that AGN acts by inhibiting noradrenaline synthesis in the rat brain and raises the possibility that an overactive noradrenergic system could contribute to depressive symptomatology, given that the therapeutic action of antidepressants reverses such overactivity via a decrease in TH expression in the LC [43]. Thus, the current results suggest that the central noradrenergic system was involved in the antidepressant effect of AGN on helpless-like behavior that persists for 21 days in rats administered repeated CORT injections. Thus, based on the present observations, a hypothesis concerning the mechanisms underlying the behavioral effects of AGN can be proposed in which the depression- and anxiety-induced behaviors occur via dysregulation of the HPA axis and the neurochemical interactions between CRF and TH in the brain.

BDNF, an important neurotrophic factor, has also been implicated in the etiology of major depression and the mechanism of antidepressant treatment [14]. A clinical study demonstrated that stress-related psychiatric disorders including depression and anxiety, are associated with reduced brain BDNF and its receptor TrkB levels [21, 44]. In a recent experiment, chronic social defeat stress or treatment with exogenous CORT caused a significant decrease in BDNF expression in the hippocampus, a brain region that may be related to the pathogenesis of depression-like symptoms [8, 21, 45]. Thus, BDNF and TrkB levels may be useful markers for antidepressant-like response [46]. To determine whether chronic CORT exposure induced changes in BDNF and TrkB expression, we analyzed BDNF and TrkB mRNAs levels in the hippocampus [11]. Consistent with these findings, we found the 3-week CORT injection decreased expression of BDNF and TrkB mRNAs in the rat hippocampus and depression-like behavior [21]. Decreased BDNF availability or reduced levels of the TrkB neurotrophin receptor could reduce BDNF signaling [47]. However, administration of AGN restored the level of BDNF and TrkB mRNAs in the hippocampus of rats subjected to repeated CORT injection, suggesting that modulation of the BDNF-TrkB neurotrophic signaling pathway may play a role in mediating antidepressant/anxiolytic actions of AGN. Also, the current results also strongly suggest a close correlation between protein and gene function in the reduced expression of BDNF in the hippocampus.

## Conclusion

In summary, the results of this study indicate the potential for AGN to effectively treat CORT-related depression and anxiety-like symptoms, possibly via modulation of the central noradrenergic system and regulation of BDNF expression. Together, these findings indicate that AGN is capable of ameliorating the complex behaviors and neurochemical responses involved in depression by modulating HPA activity. Accordingly, AGN may be a useful therapeutic agent in the development of alternative medicines for treating stress-related disorders such as depression and anxiety.

## Abbreviations

CORT: Corticosterone
HPA: Hypothalamic-pituitary-adrenal
AGN: *Angelica gigas* extract
BDNF: Brain-derived neurotrophic factor
GC: Glucocorticoid
FST: Forced swimming test
EPM: Elevated plus maze
FLX: Fluoxetine
ELISA: Enzyme-linked immunoassay
OFT: Open field test
CRF: Corticotropin-releasing factor
TH: Tyrosine hydroxylase
LC: Locus coeruleus
ABC: Avidin-biotin-peroxidase complex
DAB: Diaminobenzidine
RT-PCR: Reverse transcription**-**polymerase chain reaction
GAPDH: Glyceraldehydes-3-phosphate dehydrogenase
ANOVA: Analysis of variance
TST: Tail suspension test
